# A swift expanding trend of extracellular vesicles in spinal cord injury research: a bibliometric analysis

**DOI:** 10.1186/s12951-023-02051-6

**Published:** 2023-08-23

**Authors:** Fan Zhiguo, Wu Ji, Chen Shenyuan, Zhang Guoyou, Kai Chen, Qian Hui, Xu Wenrong, Xiao Zhai

**Affiliations:** 1 Department of Orthopedics, Shanghai Changhai Hospital, Shanghai, 200433 China; 2 Key Laboratory of Laboratory Medicine of Jiangsu Province, School of Medicine, Jiangsu University, Zhenjiang, China

**Keywords:** Bibliometric, Citation, H-index, Extracellular vesicles, Spinal cord injury, Hotspots

## Abstract

Extracellular vesicles (EVs) in the field of spinal cord injury (SCI) have garnered significant attention for their potential applications in diagnosis and therapy. However, no bibliometric assessment has been conducted to evaluate the scientific progress in this area. A search of articles in Web of Science (WoS) from January 1, 1991, to May 1, 2023, yielded 359 papers that were analyzed using various online analysis tools. These articles have been cited 10,842 times with 30.2 times per paper. The number of publications experienced explosive growth starting in 2015. China and the United States led this research initiative. Keywords were divided into 3 clusters, including “Pathophysiology of SCI”, “Bioactive components of EVs”, and “Therapeutic effects of EVs in SCI”. By integrating the average appearing year (AAY) of keywords in VoSviewer with the time zone map of the Citation Explosion in CiteSpace, the focal point of research has undergone a transformative shift. The emphasis has moved away from pathophysiological factors such as “axon”, “vesicle”, and “glial cell” to more mechanistic and applied domains such as “activation”, “pathways”, “hydrogels” and “therapy”. In conclusions, institutions are expected to allocate more resources towards EVs-loaded hydrogel therapy and the utilization of innovative materials for injury mitigation.

**Supplementary Information:**

The online version contains supplementary material available at 10.1186/s12951-023-02051-6.

## Introduction

Spinal cord injury (SCI) is a severe neurological trauma with high morbidity and mortality [1–3]. Various primary factors can cause the onset of SCI, such as compression or contusion, while secondary effects can lead to a host of complications, including neuroinflammation, microvascular damage, glial scar formation, and upregulation of inhibitory factors [4, 5]. These inhibitory factors can impede axonal extension and hinder progress towards behavioral recovery [6, 7].

At present, therapeutic effect and prognosis of SCI is limited. There are two primary treatment categories that pertain to the acute and secondary phases. During the acute phase, emphasis is placed on optimal clinical treatments that aim to prevent secondary damage by means of early surgical decompression or the administration of anti-inflammatory medicine [8–10]. And in cases of chronic phase, treatment typically involves the implementation of cell-based therapies aimed at inducing the renewal and revivification of neural tissue through the generation of neurotrophic factors, neuroprotective cytokines, anti-inflammatory agents, and stem cell transplantation [11–13].

Extracellular vesicles (EVs) have gained attention as a promising and innovative option in the field of regenerative and anti-inflammatory medicine [14–18]. EVs are a heterogeneous group of cell-derived membrane structures ranging between 100 nm and 1 µm in diameter, and EVs can be classified according to their biosynthesis or release pathway: exosomes originating in the endocytic pathway, 40–120 nm in diameter [19]. They contain a significant assortment of proteins, lipids, ribonucleic acids, and other biologically active components for intercellular communication through the dissemination of these biologically active factors [20, 21]. To our knowledge, EVs are expected to be used to treat in a variety of diseases such as myocardial infarction [22, 23], cancer [24, 25], and also SCI [26]. Studies have shown that EVs mediate cellular communication and play an important role in regulatory maintenance, tissue repair, and immune surveillance [27–30]. As a result, it is imperative to develop a comprehensive understanding of the current status and trends of EVs in SCI research.

Bibliometrics is an extensively recognized method that facilitates the analysis of the development and research patterns within a particular field [31]. It provides researchers with essential data and a holistic view of dynamic trends, aiding them in assessing existing issues, institutions, and the quantity and quality of regional publications [32]. Furthermore, bibliometrics plays a crucial role in predicting the probable future directions of research and development. It is noteworthy to mention that bibliometric findings can be instrumental in informing government policy-making authorities regarding funding decisions and other pertinent areas [33, 34]. Consequently, bibliometrics is widely employed and acknowledged as a significant tool in research evaluations. Given its numerous benefits, it is not surprising that bibliometrics continues to gain popularity among scholars and researchers across the globe.

Despite the growing interest in EVs in scientific research, there is a noticeable absence of bibliometric investigations that examine the evolution and analytical appraisal of EVs in the SCI research field. The purpose of this article is to fill this gap by assessing the global publication patterns of articles related to EVs in SCI. To achieve this goal, we have systematically organized and evaluated information on the distribution of publications stratified by country, author, journal, and impact. Moreover, we have analyzed the frequency and time of keywords in order to present the trends in the form of bibliometric maps and predict their possible directions of development in this field. By providing an in-depth analysis of the global development patterns of EVs in SCI research, this study has the potential to enhance readers’ comprehension and serve as a contemporary resource for prospective collaborative pursuits and clinical implementations.

## Materials and methods

### Literature sources and search strategy

**Fig. 1:**
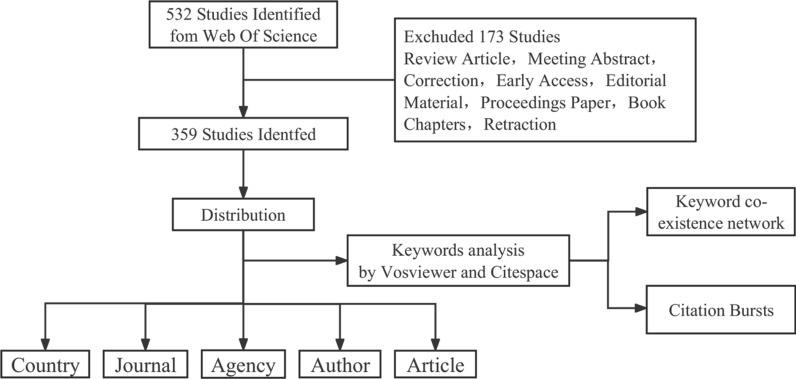
The inclusion and exclusion process of EVs in SCI research

Following the acquisition of relevant title keywords and their supplementation with mesh subject headings sourced from PubMed, we proceeded to undertake an exhaustive bibliographic search online through WoS, utilizing the search format presented below: (TS=((Spinal Cord* OR Spinal Nerves OR Myelopath*) AND (Injur* OR Regenerat* OR Trauma* OR Wound* OR Recover* OR Contusion* OR Laceration* OR Transection* OR Therapy* OR Post Traumatic))) AND TS=(Exosomes OR Endosomes OR Secretory Vesicles OR Cell-Derived Microparticles OR Exosome Multienzyme Ribonuclease Complex OR Extracellular Vesicles OR Transport Vesicles)—Time: Wed May 1, 2023, 14:54:52 GMT+0800 (CST). The articles under investigation were sourced from a period spanning January 1, 1991 to May 1, 2023. A meticulous search of available literature yielded 532 potential results which were screened, resulting in enrolling 359 papers while excluding the reviews (Fig. 1).

### Data collection and statistics

The raw data downloaded from WoS were first imported into Microsoft Excel 2019 for preliminary collation. Then two researchers (FZG and WJ) verified the assessment separately. Any discrepancies were taken for re-assessment by a third party and immediately followed by a three-way harmonization. Finally, we extracted the bibliometric parameters: the quantity of papers, frequency of citations and H-index [35]. Statistical methods include: importing the collated data into the bibliometric online analysis platform (https://bibliometric.com/) for statistical analysis on total volume; and forming mathematical fitting curves by SPSS24 (Statistical Product Service Solutions 24) to analyze the temporal trends in the number of publications published. After 2010, the cumulative number of publications in the literature had a better fitting relationship, and we used the mathematical growth model f(x) = k/[1 + a * e^(− b * x)] to fit and predict the future trend of literature accumulation [36]. Graphs were drawn using GraphPad Prism 8 (GraphPad Software Inc., CA, USA). The model equation f(x) denotes the cumulative number of papers and x represents the year. The global inflection point of the fitted curve is the point in time when the growth rate of the publication accumulation turns from positive to negative. According to the logistic growth model, when in the inflection period, it is obtained from f(x) = k/2 [37].

Using the java program VoSviewer (Leiden University, Leiden, Netherlands), a clustering analysis of keywords based on their occurrence in the title and abstract was performed [38, 39]. The frequency and interconnection of different keywords were also described by the color, size and connecting lines of the circles [40]. In addition, burst-citation analysis of keywords was performed by CiteSpace [41]. The strength was used to describe the frequency of the keywords’ occurrence [42]. The begin and end times describe the temporal distribution of the keywords. Hotspots were defined as high-frequency sub-keywords in popular scientific fields [43].

## Results

### Statistical analysis of global literature

#### Publication numbers sorted by year

**Fig. 2:**
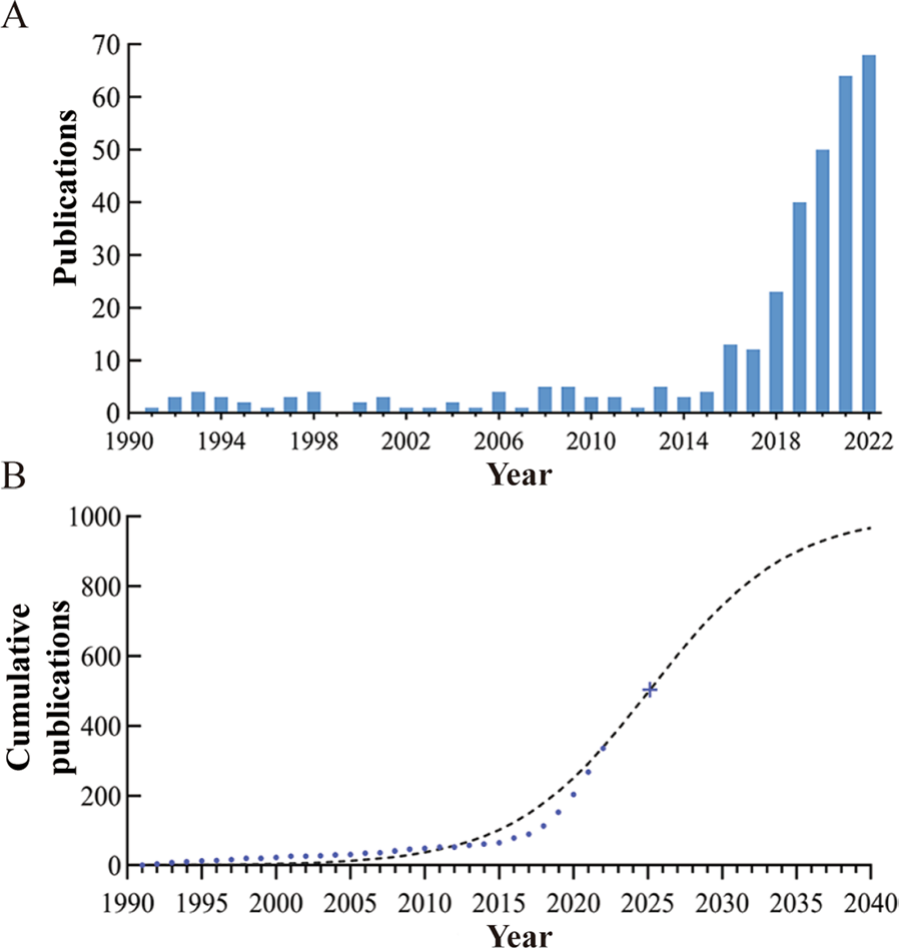
Contribution characteristics of EVs in SCI research. **A** Annual publication volume of global exosome and SCI studies. **B** Model-fitted curves of the cumulative number of publications on exosomes and SCI globally

Our search and filtering efforts encompassed 359 articles, as outlined in Additional file 2: Table S1. As demonstrated in Fig. 2A, we examined the number of articles by year, with the earliest relevant article being published in 1991. Between 1991 and 2015, publication numbers exhibited a gradual upward trend with occasional years lacking new contributions (e.g., 1999). A more substantial increase in publications occurred after 2015. Figure 2B illustrates the logistic growth curve f(x) = 1007.36/[1 + 1544.47 * e^(−0.215*(x−1991))^] for the global publication accumulation. The model's inflection point, which marks the transition from positive to negative growth rate, is projected to occur in 2025.15 as determined by the function f(x) = k/2. It is anticipated that this field will sustain a favorable development trend over an extended period.

#### Citation frequency and H-index

Based on the data gathered from WoS, the enrolled 359 publications have received a cumulative total of 10,842 citations. The mean citation frequency per article was found to be 30.2 citations, with an H-index of 56. In-depth analysis indicates that the top 100 papers, in terms of citation frequency, accounted for 75.62% of the total citations, with an average of 91.99 citations per paper. Similarly, the top 50 papers received 54.90% of total citations and had an average citation frequency of 119.04.

### Quantity and citations among different nations

**Fig. 3:**
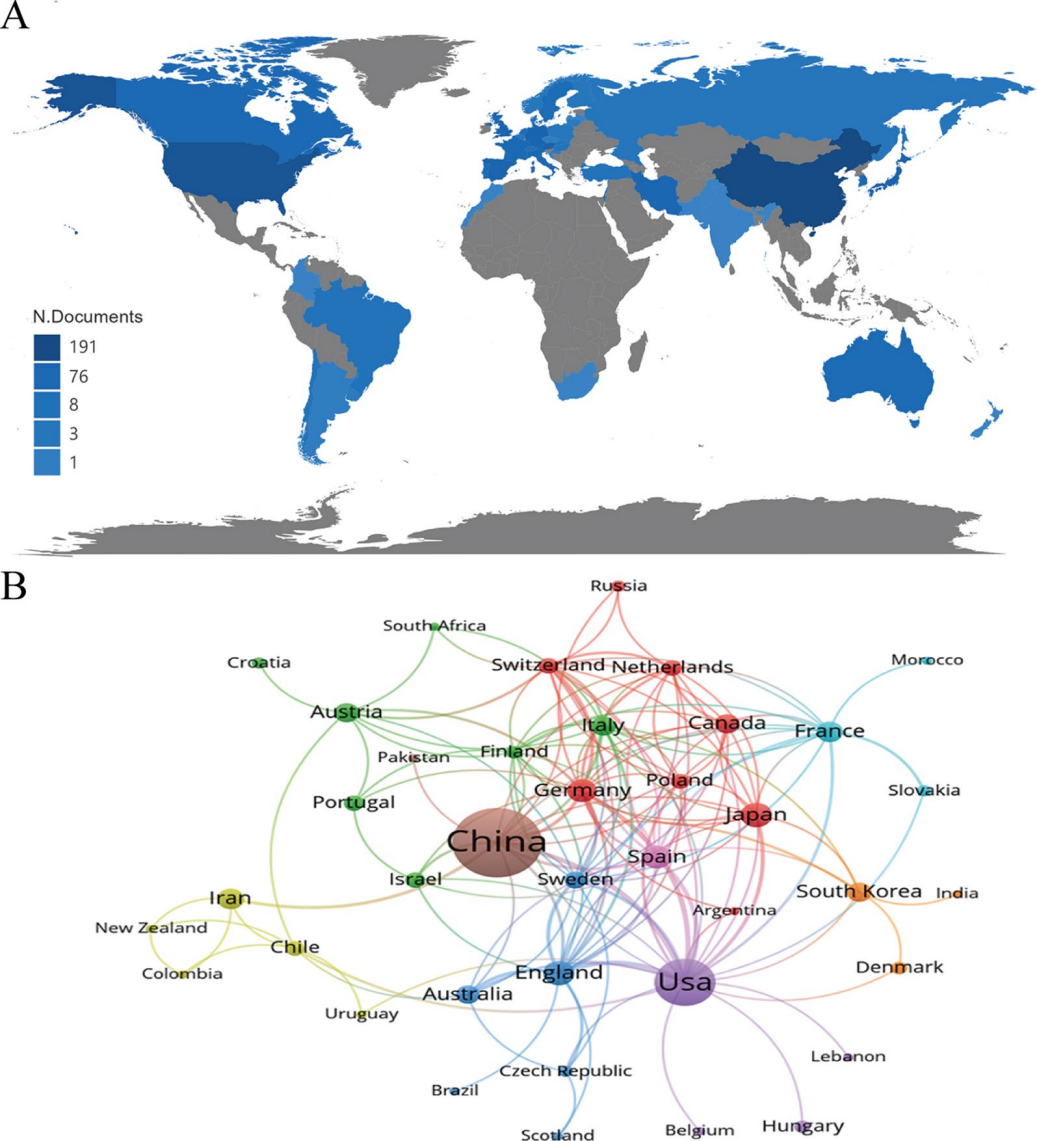
**A** Comparative map of the cumulative number of papers published in each country. **B** Cooperation networks in countries around the world

China and the USA were the most prominent countries, with the highest publications and citations. Figure 3A presents a global perspective of publications in this field, and China has the highest number of publications in this field. The USA has established a leading position in other key metrics such as total citations, average citations, and H-index (Table 1). As indicated in Fig. 3B, the USA has the highest number of interconnected targets in terms of country cooperation (25 countries, including China, the United Kingdom of Great Britain and Northern Ireland, Germany, Spain, and Sweden). It is suggested to increase efforts to strengthen international cooperation and communication.

**Table 1:** The top 10 productive countries

Country	N	Total citations	Average citations	%	H-index
China	191	4246	22.23	53.20	34
USA	76	3806	50.08	21.17	31
Japan	15	267	17.80	4.18	8
England	14	574	41.00	3.90	12
Spain	13	452	34.77	3.62	9
Germany	12	376	31.33	3.34	9
France	10	370	37.00	2.79	9
Iran	10	173	17.30	2.79	6
Australia	8	195	24.38	2.23	6
Sweden	8	247	30.88	2. 23	8

#### High-contributing journals and funding agencies

The top 10 journals in terms of publication volume published a total of 82 papers (22.84%, Table 2). Among them, *Stem Cell Research Therapy* has the highest number of publications (14), while *Journal of Neuroscience* has the highest total citations (836), average citations (76), and H-index (11).

**Table 2:** The top 10 related popular journals

Journal	N	%	Total citations	Average citations	H-index	IF-2022	JCR
Stem Cell Research Therapy	14	3.90	271	19.36	10	7.5	Q1
Journal of Neuroscience	11	3.06	836	76.00	11	5.3	Q1
Neuroscience	9	2.51	342	38.00	8	3.3	Q3
Neurochemical Research	8	2.23	69	8.63	4	4.4	Q2
Journal of Nanobiotechnology	8	2.23	174	21.75	4	10.2	Q1
Molecular Neurobiology	8	2.23	128	16.00	5	5.1	Q2
Frontiers in Neuroscience	6	1.68	266	44.33	4	4.3	Q2
Journal of Neurotrauma	6	1.68	457	76.17	6	4.2	Q2
Journal of Neuroinflammation	6	1.68	237	39.50	5	9.3	Q1
Neural Regeneration Research	6	1.68	33	5.50	2	6.1	Q2

The top 10 funding agencies are listed in Table 3. National Natural Science Foundation (NSFC) of China supported 113 studies, ranking the first (31.48%). The National Institutes of Health and the U.S. Department of Health Human Services each funded 41 researches. Within the ranking of top funding agencies, four belong to Chinese entities, while three are held by USA organizations, two by Japanese establishments and one by the EU.

**Table 3:** The top 10 related funding agencies

Funding agency	N	%
National Natural Science Foundation of China NSFC	113	31.48
National Institutes of Health NIH USA	41	11.42
United States Department of Health Human Services	41	11.42
NIH National Institute of Neurological Disorders Stroke Ninds	12	3.34
European Commission	9	2.51
Natural Science Foundation of Hunan Province	9	2.51
Natural Science Foundation of Jiangsu Province	9	2.51
Japan Society for The Promotion of Science	7	1.95
National Natural Science Foundation of Guangdong Province	7	1.95
Ministry of Education Culture Sports Science and Technology Japan Mext	7	1.95

#### Author collaboration network graph

Using VoSviewer to generate author collaboration network graphs, a total of 96 authors with more than 3 published articles formed the 16 clusters shown in Additional file 1: Fig. S1. Fifteen clusters remained after excluding single authors as clusters. Cluster 1 is primarily comprised of researchers hailing from Nanjing Medical University, with its central figure being Prof. Cai Weihua. These researchers have established collaborations with Cluster 9, led by Prof. Xu Tao. Cluster 2, led by Prof. Feng Shiqing of Tianjin Medical University, consists of researchers from both Tianjin University and Zhejiang University. The core figure of Cluster 3 is Prof. Cao Yong, affiliated with Central South University and closely collaborating with Prof. Huang Jianghu and others from Fujian Medical University of Cluster 9. Clusters 4, 5, and 6 are made up of researchers from Bengbu Medical College, Universidade de Aveiro, and Anhui Medical University, respectively. Inter-cluster cooperation is limited to Clusters 1 and 8, and clusters 3 and 9, with no collaboration discernible between the remaining clusters.

#### Characteristics of the top 10 most cited research articles

According to Table 4, the top 10 most cited articles have been referenced 2,149 times (19.82%). The article with the earliest publication date in 2013 published by Prof. Xin was also one of the most referenced, having been cited 485 times with an annual average of 44.09 citations [44]. An article by Prof. Liu’s received the highest average annual citation count of 46.75 [45]. Among the top 10, four articles were produced by academic institutions located in the USA, with three of them ranking in the top three cited articles [44, 46–48]. Additionally, four articles were attributed to Chinese academic institutions [15, 45, 49, 50], with Prof. Liu’s work ranking fourth in terms of citations [45]. Furthermore, the scholarly works produced by Prof. Guo [49], and Prof. Hervera [51] have been disseminated through international collaborations. Consequently, the articles that rank highly in terms of citations could be considered of great significance for scholars who specialize in this area of research.

**Table 4:** The top 10 most cited research papers

No	First author	Journal	Year	Citations	Citation frequency per year	Descriptions
1	Xin, Hongqi [44]	STEM CELLS	2013	485	44.09	MiR-133b is transferred by MSC-released EVs, which can promote neurite remodeling and brain plasticity by regulating genes associated with neuronal growth, such as CTGF and RhoA. It can be inferred that EVs may play potential roles in SCI by mediating gene expression regulation, neurite regeneration, modulating inflammation, immune responses, and intercellular communication pathways
2	Rebecca, P. Seal [46]	NATURE	2009	302	20.13	Dorsal root ganglion (DRG) neurons transmit sensory information to the spinal cord using the excitatory transmitter glutamate, a process that depends on glutamate transport into synaptic vesicles for regulated exocytotic release. Persistent pain caused by injury is associated with a low abundance of the vesicular glutamate transporter VGLUT3 expressed by a small subset of cells in the DRG
3	Veronica, J. Tom [47]	J NEUROSCI	2004	206	10.31	Time-lapse movies demonstrated that dystrophic endings after SCI continually send out membrane veils and endocytose large membrane vesicles at the leading edge, which were then retrogradely transported to the rear of the “growth cone”
4	Liu, Wei [45]	J NEUROINFLAMM	2020	187	46.75	Hypoxia preconditioning represents a promising and effective approach to optimize the therapeutic actions of MSC-derived EVs. And a combination of MSC-derived EVs and miRNAs may present a minimally invasive method for treating SCI
5	Guo, Shaowei [49]	ACS NANO	2019	168	33.61	EVs therapy promotes recovery from SCI: MSC-Exo, administered intranasally, can cross the blood–brain barrier and migrate to the injured spinal cord area. ExoPTEN loaded in MSC-Exo reduces PTEN expression, enhances axonal growth and neovascularization, decreases microgliosis and astrogliosis, improves structural and electrophysiological function, and significantly promotes functional recovery in rats with complete SCI
6	Huang, Jianghu [50]	J NEUROTRAUM	2017	167	23.86	Systemic administration of MSCs-EVs attenuated cell apoptosis and inflammation, promoted angiogenesis, and promoted functional recovery post-SCI, suggesting that MSCs-EVs hold promise as a novel therapeutic strategy for treating SCI
7	Gimona, Mario [169]	INT J MOL SCI	2017	163	23.29	In this article, they discussed the requirements for manufacturing, safety, and efficacy testing of EVs along their path from the laboratory to the patient. They also deliberated the rationale for testing MSC-EVs in selected diseases with an unmet clinical need such as critical size bone defects, epidermolysis bullosa and SCI
8	Hervera, Arnau [51]	NAT CELL BIOL	2018	161	26.83	ROS promote axonal retraction and degeneration, but they are also necessary for axonal regeneration and recovery after SCI. EVs contribute to spinal cord regeneration after injury by activating the NOX2-PI3K-p-Akt signaling pathway
9	Sun, Guodong [15]	MAT SCI ENG C-MATER	2018	155	25.83	HucMSC-derived EVs can promote SCI healing by suppressing inflammatory response. They modulate the polarization of inflammatory cells and downregulate inflammatory factors, improving functional recovery. These findings offer a new perspective and therapeutic strategy for SCI treatment
10	Vaccari, J. De Rivero [48]	J NEUROCHEM	2016	155	19.83	EVs act as carriers to deliver siRNA and inhibit inflammasome activation, thereby suppressing neuroinflammatory responses following SCI. This provides a novel therapeutic approach for treating inflammation and cellular damage induced by central nervous system injuries

### Keyword analysis of global research

#### Keyword co-existence network analysis

**Fig. 4:**
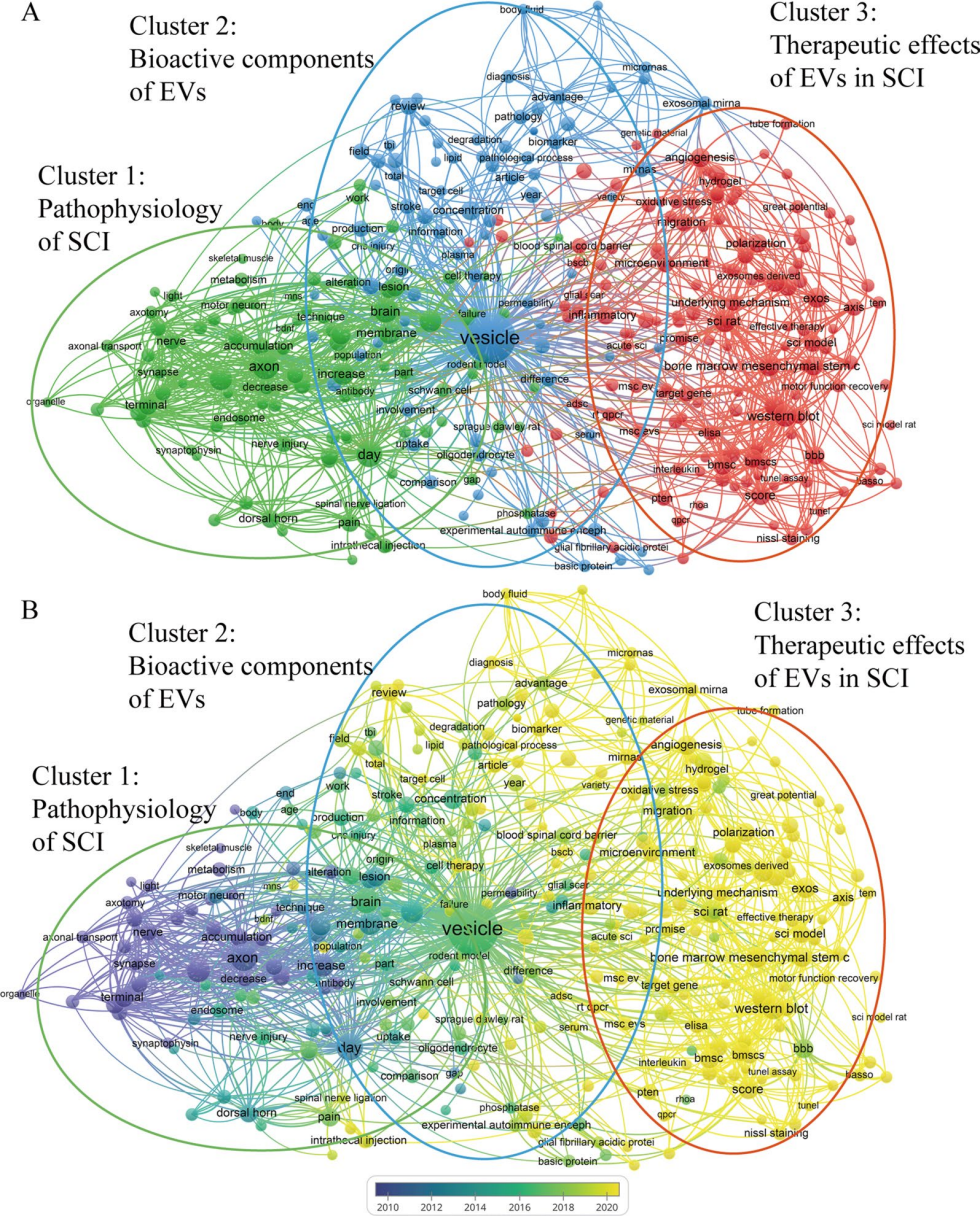
Keyword co-occurrence network and keyword evolution over time. **A** Keyword co-occurrence network is divided into 3 clusters of different colors according to the evolution of the research hotspots. **B** The color of a keyword indicates the average publication time of articles containing that keyword

The keywords were filtered from the existing data, and a total of 333 words were obtained, which could be divided into 3 clusters (Fig. 4A). Cluster 1: “Pathophysiology of SCI” denotes green, Cluster 2: “Bioactive components of EVs” represents blue, and Cluster 3: “Therapeutic effects of EVs in SCI” is indicative of red. The magnitude of the keyword circle within each cluster is commensurate with the frequency of its appearance (Additional file 3: Table S2). The main terms comprising cluster 1 are “axon” [52, 53], “day” [54, 55], “immunoreactivity” [56, 57] and “glial cell” [58, 59]. Meanwhile, cluster 2 is characterized by the core keywords “vesicle” [60–62], “source” [63, 64], “miRNAs” [65, 66] and “biomarker” [67–69]. Lastly, cluster 3 is distinguished by “bone marrow mesenchymal stem cells” [70–72], “lipopolysaccharide” [73, 74], “angiogenesis” [75–77], “apoptosis” [78, 79], “polarization” [80, 81], “migration” [82, 83], “hydrogel” [84, 85], “cell communication” [86, 87], and “mechanism” [88, 89] as its core keywords.

**Fig. 5:**
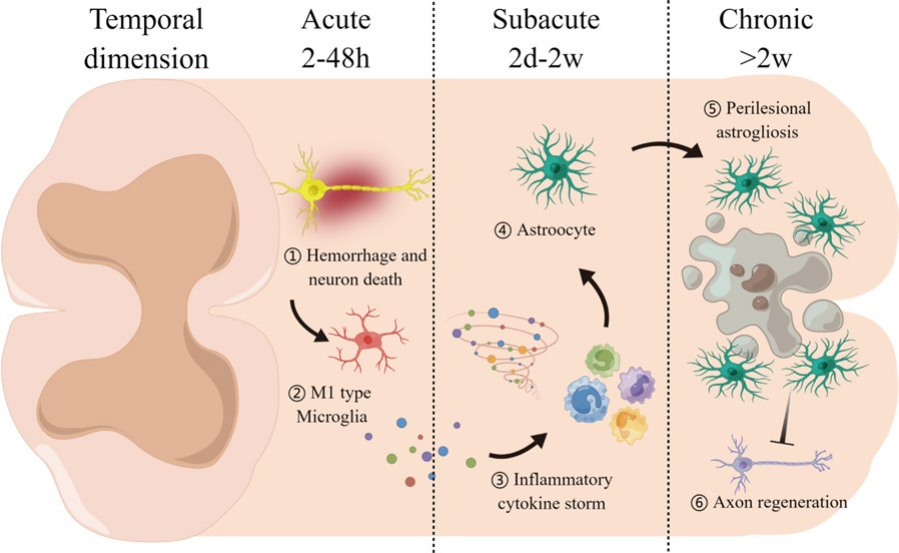
Schematic illustration of Cluster 1: “Pathophysiology of SCI”. The temporal window, which can be counted by day, is of paramount importance in the treatment of SCI. The initial mechanical trauma to the spinal cord initiates a secondary injury cascade in the acute phase (2–48 h): oedema, hemorrhage, ischemia, neuron death, and activation of M1 type microglia. The activation of innate immunity by M1 type microglia leads to the subacute phase (2d–2w): persistent inflammatory cell infiltration and cytokine storm, glia scar formation by astrocytes. Chronic SCI (> 2w): glia scar maturation, cyst formation, and inhibition of axon regeneration. Created with Figdraw.com

**Fig. 6:**
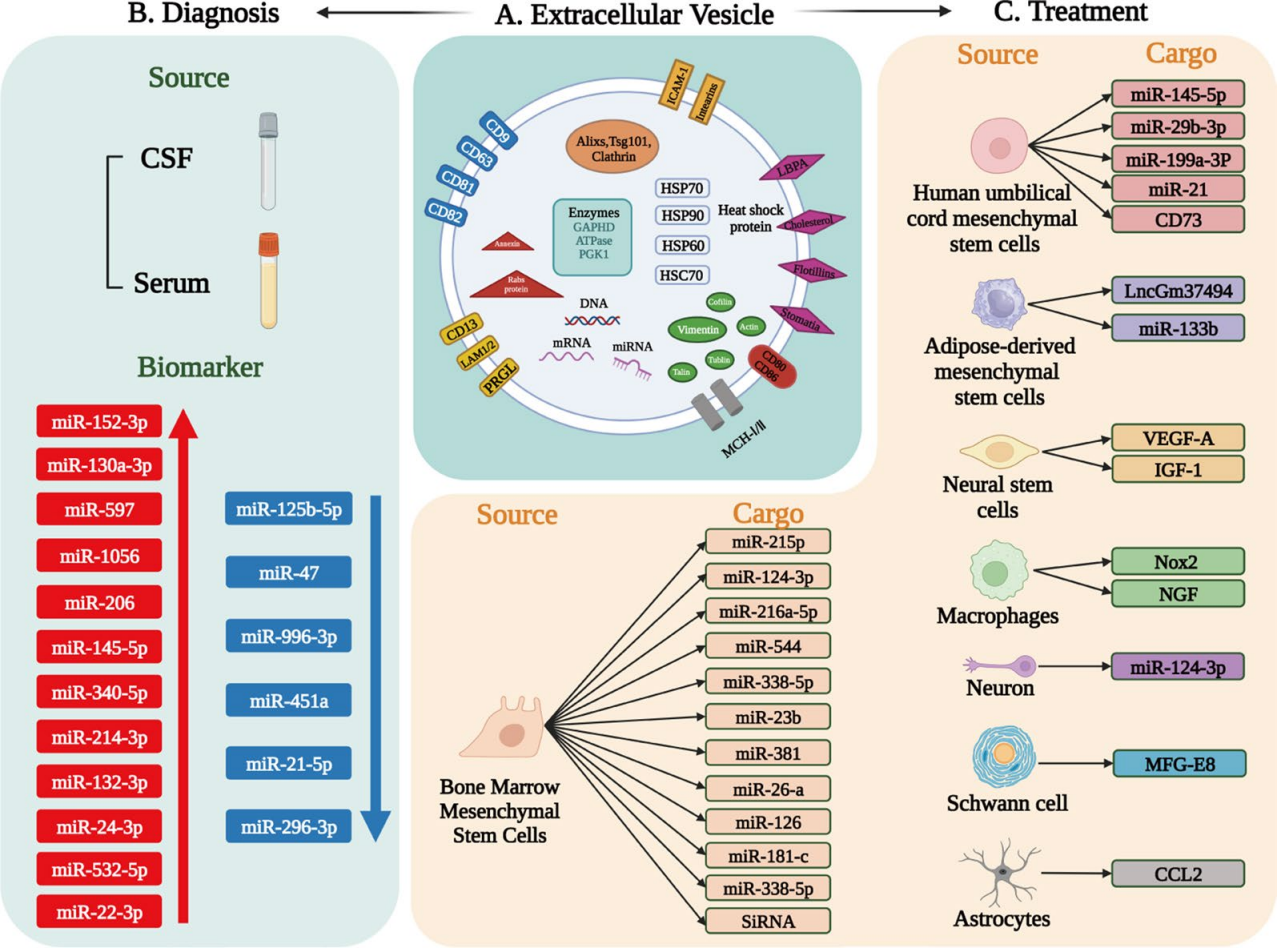
Schematic illustration of Cluster 2: “Bioactive components of EVs”. **A** Structure, bioactive components, and distribution in the EV. (Reprinted with permission from Ref. [168], Copyright 2023, Frontiers.) **B** EVs present in blood and cerebrospinal fluid, demonstrate stability while exhibiting changes in the concentration of bioactive compounds that they carry or discharge. These changes are linked to the pathological state of SCI, making them a potential diagnostic tool and reliable biomarker for identification. **C** EVs derived from diverse cells are known to contain a multitude of bioactive components that can activate distinct molecular pathways involved in the process of axonal regeneration and repair. Created with BioRender.com

**Fig. 7:**
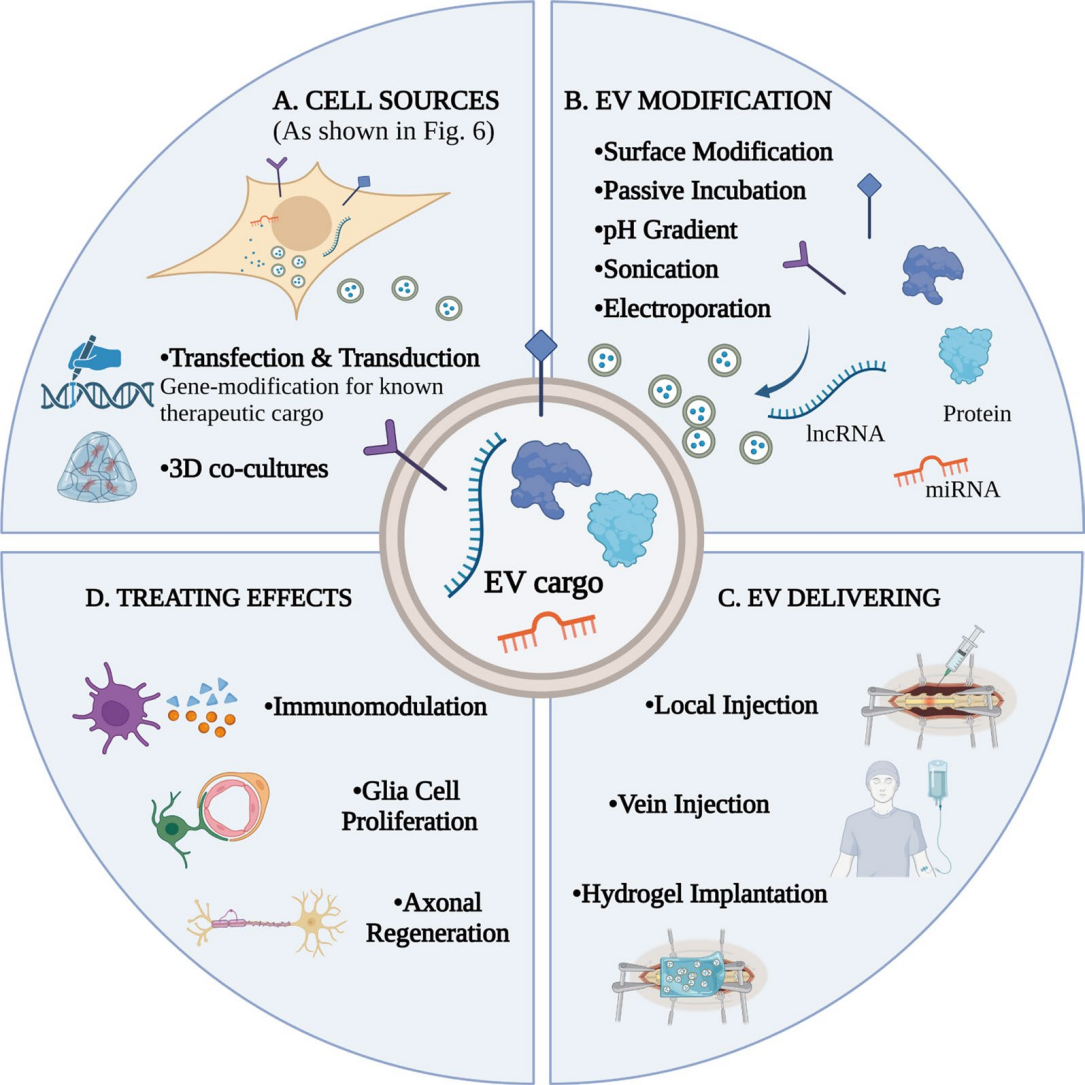
Schematic illustration of Cluster 3: “Therapeutic effects of EVs in SCI”. **A** EV producer cells are summarized in Fig. 6. Gene-modification and 3D co-cultures are regularly used to further enhance cellular loading of known biological mediators. **B** For functional cargo makeup on EV surface, techniques are utilized such as surface modification, passive incubation, pH gradient, sonication, electroporation. **C** For clinical use of engineered EV, local injection and vein injection are effective ways, and hydrogel implantation can achieve sustained controlled release. **D** EVs have been reported of therapeutic effects such as immunomodulation, glia cell proliferation, and finally axonal regeneration. Created with BioRender.com

VoSviewer marks the keywords in the figure with different colors depending on the average appearing year (AAY) of the keywords. The purple keywords appeared earlier than the blue and yellow ones (Fig. 4B). The shift from purple to yellow delineates the progression of keyword advancement. Figures 5, 6, 7 provides a comprehensive overview of the focal point of the researchers’ investigation, which encompasses keywords with 3 clusters, including “Pathophysiology of SCI”, “Bioactive components of EVs”, and “Therapeutic effects of EVs in SCI”.

#### Citation bursts analysis

**Fig. 8:**
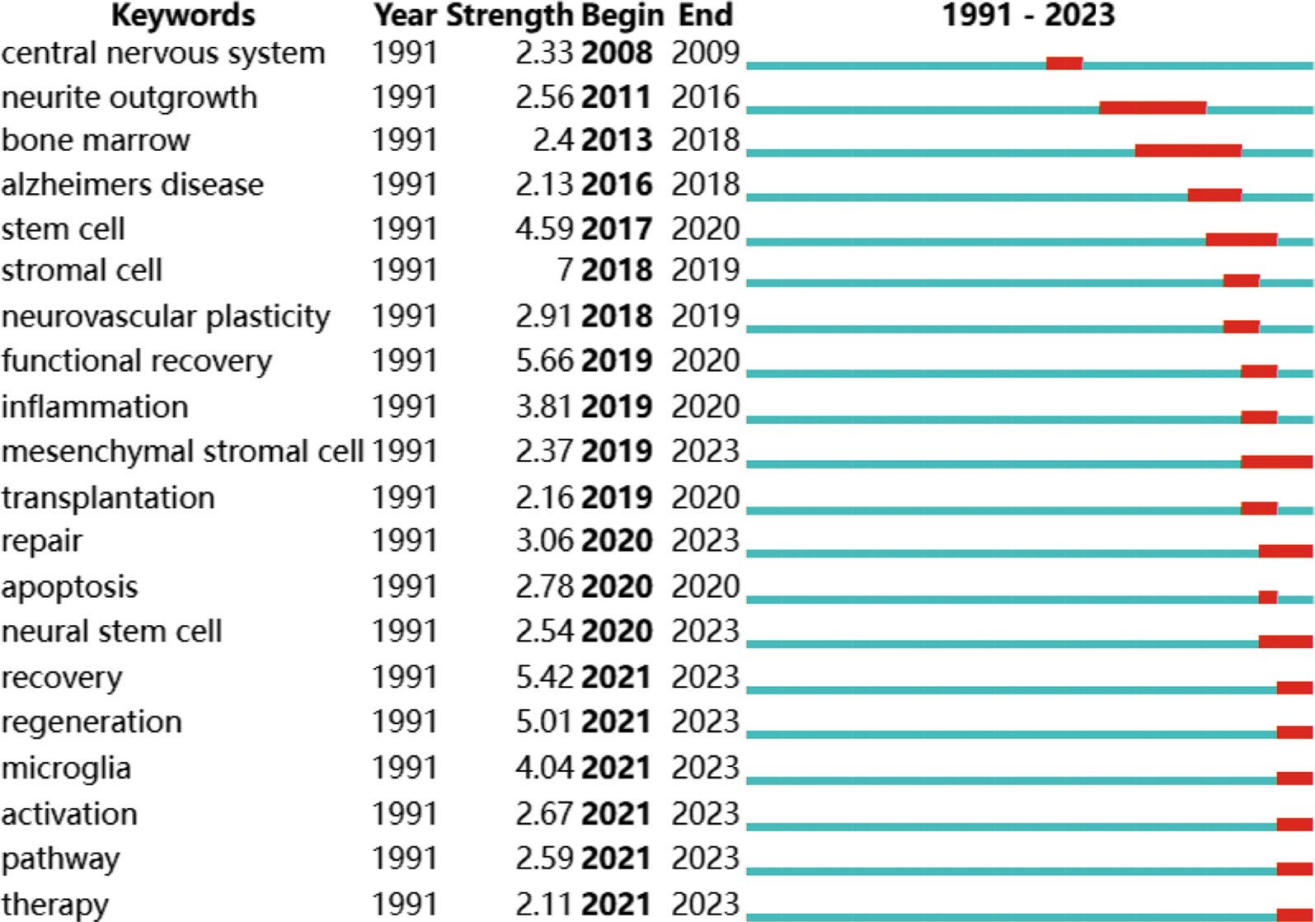
Top 20 keywords with the most robust citation bursts

Although VoSviewer effectively displays the co-occurrence status of keywords, it presents limitations in illustrating changes in keyword prominence. It solely visualizes the annual activity of publications and neglects to indicate the start and end time and sudden bursts of keywords. To address this issue, we utilized CiteSpace software to extract the citation burst for all keywords, with a particular focus on the top 20 keywords. The most significant citation burst belongs to “stromal cell”. Notably, since 2020, the keywords “mesenchymal stromal cell”, “repair”, “neural stem cell”, “recovery”, “regeneration”, “microglia”, “activation”, “pathway”, and “therapy” have been more prominently concentrated, indicating promising developments (Fig. 8).

## Discussion

### Research of EVs in SCI is currently garnering significant attention and interests

In recent years, there has been a noteworthy escalation in scholarly publications concerning EVs in SCI. Additionally, the field has undergone a remarkable upsurge in worldwide publications since 2015. According to the logistic growth model, the global inflection point might occur in 2025, thus this field will still have a strong momentum in the next a few years. It seems to be attributed to the 2013 Nobel Prize in Physiology or Medicine, which was conferred upon three distinguished scientists, James E. Rothman [90, 91], Randy W. Schekman [92, 93], and Thomas C. Sudhof [94, 95], for their contributions to unraveling the mechanisms of transport and regulation of EVs. It has brought EVs to widespread attention and has attracted the participations and supports from research institutions in various countries. Accordingly, the number of studies on the relationship between EVs and SCI is gradually increasing.

Research initiatives have already been undertaken in the area of EVs and SCI in several countries, with China and the United States leading the charge. Of the 359 articles retrieved, 191 (53.20%) were published in mainland China, and 76 (21.17%) were in the USA. In particular, mainland China has an explosive growth in production since 2015, while the USA has a continuously steady growth since 1991. In terms of inter-country cooperation, the network demonstrates universal collaboration comprises a significant fraction from the USA (Fig. 3B), while Chinese study mostly occurred among domestic institutes. In addition, the top 10 most prolific authors in this field are all from China. Prof. Cai and Prof. Liu from China are leaders in the field and they might remain leading the development of the field. Theoretically, these top scholars are the optimal choices for cooperation and communication.

Within this domain, there are certain journals and funds that worth the attention of researchers. The journals listed in Table 2, such as *Stem Cell Research Therapy*, *Journal of Neuroscience*, *Neuroscience*, and *Journal of Nanobiotechnology*, are probably the core journals in this field, hence submitting relevant papers to these journals is recommended. Researchers also need to pay more attention to the latest articles published in these journals. In addition, the funding agencies listed in Table 3, such as the NSFC, the NIH and the U.S. Department of Health Human Services, are worthy choices for researchers to apply for.

### Research focus shifts from pathophysiological mechanisms to innovative materials

The combination of bibliometrics and visual mapping has become an effective way of quantitatively and systematically assessing trends in a particular field, and can also predict possible research directions. In this study, keywords were divided into 3 clusters: “Pathophysiology of SCI” (Cluster 1, green), “Bioactive components of EVs” (Cluster 2, blue), and “Therapeutic effects of EVs in SCI” (Cluster 3, red). And in Fig. 4B, it shows that interests of researchers' investigation gradually shift from Cluster 1 to Cluster 2, and then Cluster 3.

#### Cluster 1: “pathophysiology of SCI”

As shown in the Cluster 1, the main terms are “axon”, “day”, “immunoreactivity”, and “glial cell”. This cluster of terms is summarized as pathophysiological mechanisms of SCI, encompassing the development of the injury, influences of glial cells, as well as the importance of axonal regeneration (Fig. 5). Further studies for an in-depth understanding of the mechanism of pathophysiology in SCI may be beneficial for the development of novel therapeutic strategies.

Firstly, gaining a comprehensive understanding of the temporal trajectory of SCI is essential for devising effective treatment strategies that can mitigate the negative impact of this debilitating condition [96, 97]. The initial mechanical trauma to the spinal cord initiates a secondary injury cascade in the acute phase (2–48 h), which is featured with oedema, haemorrhage, ischaemia, neuron death, and activation of M1 type microglia. Subsequently, the activation of innate immunity by M1 type microglia leads to the subacute phase (2d–2w), which is manifesting with persistent inflammatory cell infiltration and cytokine storm, glia scar formation by astrocytes. Moreover, in the long time of chronic SCI (> 2w), the glia scar maturation and the cyst formation finally inhibited of axon regeneration [98]. The temporal dimension of this condition refers to the critical period during which the injury occurs and subsequently evolves [99]. For examples, endothelial-specific expression of plasmalemmal vesicle associated protein-1 (PV-1) showed expression as early as 1-day post-SCI, with levels decreasing by 14 days, which was associated with microvessels in the injury epicenter and penumbral zone, with the time course and distribution correlated with progressing peripheral inflammatory cell infiltration [100]. Up to date, single-cell transcriptomic analyses provide a comprehensive mapping of cellular/molecular pathological changes along the temporal axis after SCI [101]. As a result, the manifestation and progression of SCI represents a complex process that is influenced by a multitude of factors [101, 102]. And this timeframe is of paramount importance, as it plays a crucial role in determining the extent of the damage and the potential for recovery.

Secondly, it is important to note that glial cells play a crucial role in shaping the microenvironment surrounding axons [103]. Glial cells have both beneficial and detrimental effects on the recovery process of axons, when the level of immunoreactivity is raised. The heightened immunoreactivity in glial cells can be observed as soon as 1 day after the injury and remains present for an extended period of time [104]. In addition, the inciting traumatic incident and its resulting neurophysiological disturbance elicit immune, endocrine, and multisystemic dysregulation, subsequently impacting the patient's mental state and overall health [105]. Therefore, it is essential to understand the immunoreactivity that arises after such injuries in order to develop effective treatments and therapies. Gaining insight into immunoreactivity after SCI will facilitate the development of treatments that can aid in the recovery process of those suffering from neurological disorders [106].

Finally, regeneration of axons is a primary focus and challenge in the treatment of SCI [107]. Axons have a crucial function to transmit messages between nerve cells, and their proper functioning is necessary to maintain the sensation and movement of the human body [108]. However, SCI can cause extensive damage to axons, leading to a significant reduction in a patient's ability to feel or move [109, 110]. Therefore, the discovery of effective methods to stimulate the regeneration of axons within the spinal cord is of utmost importance to researchers and clinicians. This research area holds immense potential to enhance the quality of life of SCI patients, and as such, this field of study should be given due attention and investment [111–113].

#### Cluster 2: “bioactive components of EVs”

Cluster 2 is characterized by the core keywords “vesicle”, “source”, “miRNAs” and “biomarker”. In this cluster, bioactive components of EVs can be summarized for the usage of diagnosis and treatment (Fig. 6).

On one hand, the identification of a biomarker for SCI diagnosis could be achieved by analyzing the contents of EVs. These vesicles were found to carry specific microRNAs (miRNAs) [114], lncRNAs [3] and proteins that reflect the physiological state of the injured tissue [69]. From analyzing SCI-induced changes in circulating plasma EVs, it resulted in multifaceted changes in total plasma EVs at 1d post-injury including a decrease of miR-206, miR-145-5p, miR-34c-5p, miR-214-3p, miR-132-3p, miR-24-3p, miR-532-5p, and miR-22-3p, and an increase of miR-125b-5p, miR-47, miR-996-3p, miR-451a, miR-21-5p, miR-296-3p [60, 115]. By pinpointing the biomarkers present in these vesicles, it could be possible to establish a diagnosis for spinal cord injury with a higher degree of accuracy, and ultimately facilitate the development of novel therapies [116–119].

On the other hand, EVs derived from various sources can be useful for treating SCI. EVs exert their effects through enhancing cell communication, promoting regeneration, and reducing inflammation in injured tissues, which inherited from the parent cells [107, 120–126]. The source of EVs should be carefully considered before clinical applications as it may have an impact on their therapeutic efficacy. The sources of EVs such as mesenchymal stem cells [26, 49, 127–135], neural stem cells [136–139], and Schwann cells [52, 140, 141] have shown promising results in promoting regeneration and functional recovery. And for therapeutic cargos, the bibliometric data in this study showed that “miRNAs” appeared 32 times [65, 142, 143], and the most representative one is the miR-216a-5p studied by Prof. Liu [45]. Therefore, research in this area holds significant promise for the development of more effective diagnostic tools and treatments for SCI patients.

#### Cluster 3: “therapeutic effects of EVs in SCI”

Figure 4A illustrates the distinguished keywords in Cluster 3 as “bone marrow mesenchymal stem cells”, “angiogenesis”, “apoptosis”, “polarization”, “migration”, and “hydrogel”. The focus of this particular cluster is to elucidate the concept of “therapeutic effects of EVs in SCI”. To provide a comprehensive understanding of this topic, we intend to delve into four distinct facets: the use of EVs producer cells, functional cargo makeup on EVs surface, EVs delivering, and EVs treating effects (Fig. 7). By exploring these areas, we aim to present a well-rounded perspective on the potential benefits and limitations of EVs-based therapies for SCI.

Initially, EVs producer cells are summarized in Fig. 6. It is imperative to emphasize the importance of MSCs regarding the application of EVs. Scientific researches have demonstrated that EVs originating from MSCs, particularly those from bone marrow MSCs, possess a remarkable ability to modulate the immune system, effectively controlling the inflammatory damage that results from SCI [144–148]. Additionally, MSC-derived EVs have been shown to stimulate angiogenesis, promote the proliferation of oligodendrocytes, facilitate axonal regeneration, remyelination, and mitigate fibrosis, thereby promoting the healing of neurons [107, 149–153]. These findings, supported by numerous studies and publications, highlight the potential of EVs derived from MSCs as a critical therapeutic tool for SCI. Additionally, to enhance cellular loading of known biological mediators, gene-modification and three-dimensional co-cultures are regularly used. Studies showed that EVs derived from CD73 modified human umbilical cord MSCs ameliorated inflammation after SCI [26], and EVs enriched with miR-219a-5p using a gene-modified HEK293T cell line improved experimental autoimmune encephalomyelitis [154]. And three-dimensional microenvironments provide increased control over spatial distribution of materials (e.g. bioactive components, cells, drug depots, etc.) that might improve the therapeutic effects of EVs [155, 156]. As a result, cells source and cells microenvironments are critical determinant of EV bioactivity.

EVs can be further modified to act as nanodrug carriers and have shown therapeutic potential for central nervous system disorders. Modified EVs function through the nucleic acids (mRNA, microRNA, non-coding RNAs) and proteins they transport [19, 65]. Considering that the standard loading of endogenous non-coding RNA into EVs can be as low as a single functional copy of a specific miRNA per vesicle, this may not result in a significant alteration of gene expression in the intended recipient cells [157]. Therefore, it becomes imperative to increase the potency of EVs by enhancing their ability to carry and deliver therapeutic cargo. Several techniques are utilized for functional cargo makeup on EV, such as surface modification, passive incubation, pH gradient, sonication, and electroporation [115, 158].

In addition, it is noteworthy that hydrogels have been established as a suitable medium for delivering EVs to injury sites. The use of hydrogels holds immense value as it enables the targeted delivery of therapeutic agents to specific regions of the injury site. Additionally, the sustained and controlled release of these agents from hydrogels further enhances the potential effectiveness of treatment. Relevant scholarly sources [85, 106, 120, 131, 149, 159, 160] have also supported this notion. Consequently, the utilization of EVs holds immense promise for the development of therapeutic interventions aimed at improving outcomes for individuals with SCI [85].

Finally, EVs-mediated signaling targets CNS-specific regenerative processes after SCI, such as immunomodulation, remyelination, glial scar formation, and axonal regeneration. It is due to their ability to cross the blood-spinal cord or blood–brain barrier, as evidenced in studies [161–165]. Profiling of EVs associated cargo can be used to identify and validate any critical bioactive components that may mediate therapeutic benefit.

## Strengths and limitations

In this study, the combination of bibliometrics and visual analysis can provide readers with systematic information on the study of EVs in SCI, helping them to easily access the progress and trends of research in this field. Additionally, our work can provide potential partnerships as well as reliable information for scientists and funding agencies, as it based on different regional, institutions, and researcher stratification.

However, there are some limitations in this study. First, only English papers from the WoS database were included, and papers in other languages failed to be included in the study. The concept of Science Citation Index is based on Bradford's law in bibliometrics, which can be used to define a core set of journals or publications, and the journals included in the WoS Science Citation Index Extended (SCI-E) database are described as world-leading journals due to their rigorous selection process [166]. Thus, publications included in WoS can be representative of research in the discipline. Second, new papers published after the search date were not included in the study because the database was kept open [167]. Moreover, the growth trend of the paper may last longer than the mathematical model predicts.

## Conclusion

Since 2015, there has been a notable increase in the number of publications in the field of EVs in SCI, with Mainland China and the United States emerging as high productivity nations. As research progresses, there has been a shift in focus towards investigating mechanisms of injury mitigation and the integration of innovative materials. It is expected that institutions will allocate greater resources towards the advancement of EVs-loaded hydrogel therapy. To achieve substantial advancements in this area, it is recommended to designate emphasis on key topics such as “glial cells”, “neuroregeneration”, “angiogenesis”, “cell scorching”, “cell communication”, and “hydrogels”.

### Supplementary Information

**Additional file 1: Figure S1.** Collaborative network of authors.

**Additional file 2: Table S1.** Details of all 359 exosomes and spinal cord injury research papers.

**Additional file 3: Table S2.** Detailed information of all keywords in VoSviewer, arranged in ascending order of clustering and descending order of occurrences.

## Data Availability

The dataset supporting the conclusions of this article is included within the article (Additional files 1, 2, 3).

## Abbreviations

SCI: Spinal cord injury
WoS: Web of Science
AAY: Average appearing year
USA: United States of America
UK: The United Kingdoms of Great Britain and the Northern Irelands
EU: European Union
NSFC: National Natural Science Foundation of China
NIH: National Institutes of Health
MSC: Mesenchymal stem cell
BMSC: Bone mesenchymal stem cell
hMSC: Human placental amniotic mesenchymal stem cell
SCI-E: Science Citation Index-Expanded
