# Supplementary figures and images for: A swift expanding trend of extracellular vesicles in spinal cord injury research: a bibliometric analysis
Source: J Nanobiotechnology. 2023 Aug 23;21:289. doi: 10.1186/s12951-023-02051-6 (PMC10463993; doi:10.1186/s12951-023-02051-6)

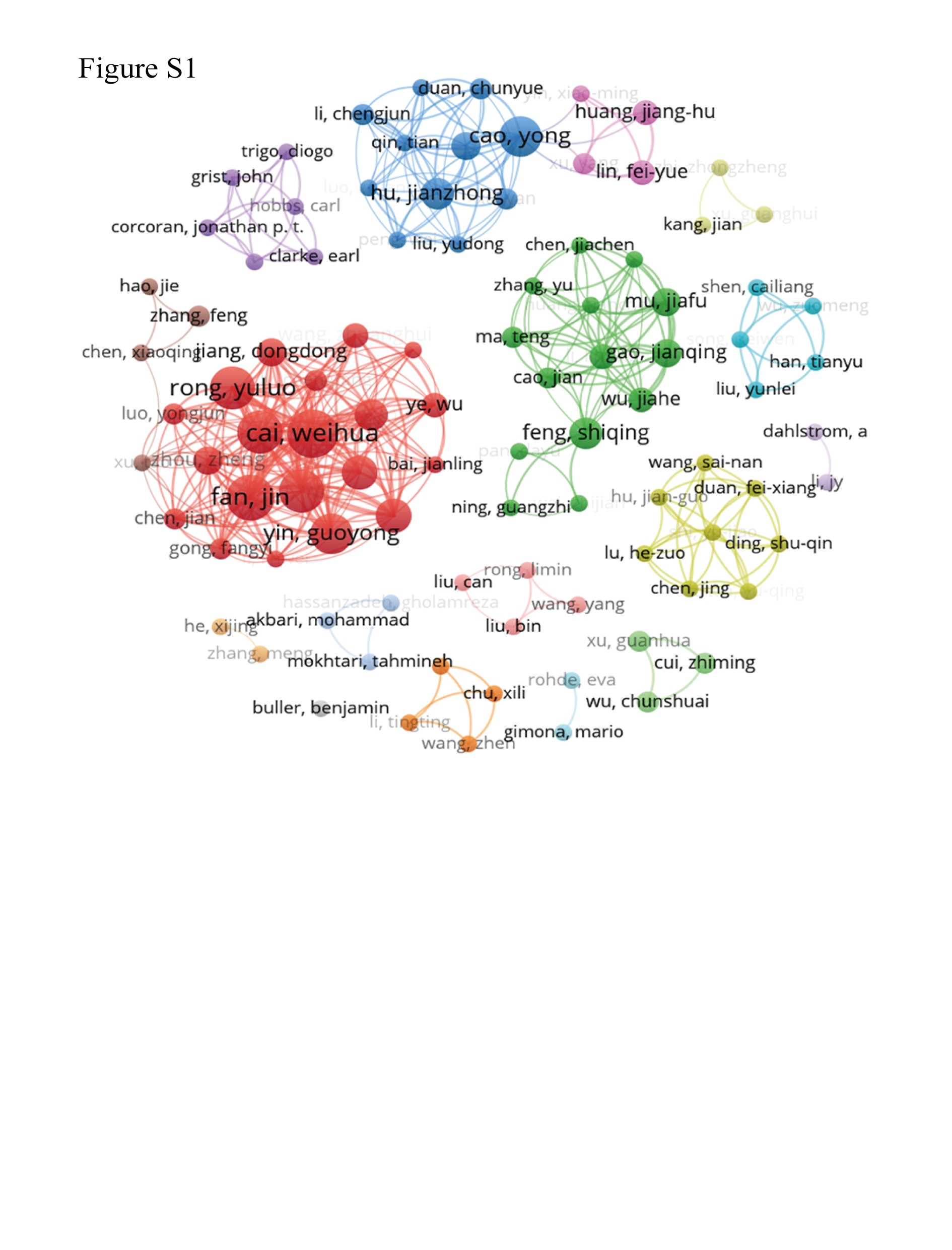

Supplement: Supplementary file 1 — Additional file 1: Figure S1. Collaborative network of authors. [file 12951_2023_2051_MOESM1_ESM.tif]
